# Effects of yeast and dried kratom leaves (*Mitragyna speciosa* [Korth] Havil.) supplementation on digestibility, rumen fermentation, blood metabolites and nitrogen balance in goats

**DOI:** 10.5713/ab.23.0153

**Published:** 2023-12-11

**Authors:** Soklin Va, Chanadol Supapong, Pin Chanjula

**Affiliations:** 1 Animal Production Innovation and Management Division, Faculty of Natural Resources, Hat Yai Campus, Prince of Songkla University, Songkhla 90110, Thailand; 2 Department of Animal Science, Faculty of Agriculture, Rajamangala University of Technology Srivijaya, Nakhon Si Thammarat 80240, Thailand

**Keywords:** Goat, *Mitragyna speciosa* (Korth) Havil., Rumen Fermentation, Yeast

## Abstract

**Objective:**

The objective of the experiment was to study yeast supplementation (yeast, Y) and dried kratom leaves (DKTL) on the digestibility, ruminal fermentation, blood metabolites and nitrogen balance in goats.

**Methods:**

Four of 7 to 8 months old male crossbred (50% Thai Native-Anglo Nubian) goats with average liveweight 20±0.13 kg were randomly assigned according to a 2×2 factorial arrangement in a 4×4 Latin square design to receive four diets *ad libitum* basis. The study investigated the effects of two levels of yeast (Y) supplementation (Y, 0 and 0.5g/kg dry matter [DM]) along with two levels of DKTL supplementation (DKTL, 0 and 4.44g/kg DM). The experimental groups were as follows: T1 = control group with 0Y+0DKTL, T2 = 0Y+4.44 DKTL, T3 = 0.5Y+0DKTL, and T4 = 0.5Y+4.44 DKTL.

**Results:**

The results showed that there were no interactions between Y levels and DKTL levels with respect to total DM intake, but there were significant effects (p<0.05) by levels of Y; goats receiving 0.05 g/kg DM Y had higher than goats fed 0.0 g/kg DM on average (kg/d). A percentage of body weight (% BW) and grams per kilogram of metallic weight (g/kg w^0.75^) had no influence on yeast levels and DKTL, but there was a difference (p<0.05) by yeast level Y at 0.5 g/kg DM, being higher compared to the non-supplemented group. Apparent digestibility coefficient of nutrition in the form of (DM, organic matter, crude protein, neutral detergent fiber, and acid detergent fiber) was an increased trend in the Y-level complementary group at 0.5 g/kg DM and DKTL at 4.44 g/kg DM, respectively. Protozoa populations decreased in the group receiving Y levels at 0.5 g/kg DM and DKTL levels at 4.44 g/kg DM when compared to group T1. The acetic acid concentration and methane gas generation decreased (p<0.05) in the group receiving Y levels of 0.5 g/kg DM and DKTL levels of 4.44 g/kg DM, while the amount of propionic acid increased (p<0.05).

**Conclusion:**

Effects of feeding combinations of Y and DKTL supplementation on feed showed no interaction effect (Y×DKTL) on feed intake, rumen fermentation, bacterial and fungi population. The effect on protozoal populations was lower in the group that was supplemented with DKTL at 4.44 g/kg DM related to synthetic CH_4_ was reduced.

## INTRODUCTION

Feed additives are products used in animal nutrition for the purpose of enhancing the health and performance of the animals. The discovery of antibiotics in the United States in the late 1940s led to their early use in diets [1]. The advantages of antibiotics are increased feed intake, nutritional digestibility, endocrine and immunological responses, and intermediate nutrient metabolism [2]. However, the use of synthetic antibiotics as feed additives is currently prohibited due to the development of bacterial resistance to them because of their increased presence in food chains with residues of these chemicals in meat and milk. Much effort has been put into creating substitute feed additives in their place.

The most common yeast additive used in ruminant diets is obtained from cultures of *Saccharomyces cerevisiae*. Yeast is added to feed to enhance the activity of beneficial microbes in fermentation, reduce energy and nutrient losses thus improve the digestibility of nutrients and production potential of the animals [3].

Yeast is one of the probiotics commonly applied in ruminant nutrition research and production. Supplementation with yeast may improve feed intake and milk production in dairy cattle [4]. Yeast has potential to enhance fibre digestion in the rumen by aiding the growth and reproduction of anaerobic rumen microbes, especially most cellulolytic bacteria [5].

Herb use is increasing in popularity because it offers numerous benefits and has no negative side effects [6]. *Mitragyna speciosa* (Korth) Havil. is an herbaceous plant found mostly in Thailand’s southern regions, which is a native Southeast Asian tropical tree known as kratom in Thailand [7]. *Mitragyna speciosa* (Korth) Havil. has a mitragynine of 4.14%, total condensed tannin (CT) and saponin (SP) are 8.28 and 5.21, respectively. Flavonoid content of 11.24% according to a previous study [7].

Both a stimulant and a depressive for the central nervous system, kratom has been labeled. Plant secondary metabolites can have an impact on animal health, performance, and product quality [8]. Similar to this, Chanjula et al [7] revealed that dried kratom leaf (DKTL) supplementation may be a fantastic substitute supplement for goat feed when compared to the control diet. DKTL supplementation can be used as dietary effect on body weight (BW), average daily gain, feed conversion ratio, carcass composition, meat pH, or meat color (p>0.05), either. In conclusion, DKTL supplementation can enhance the quality of goat meat. Up to now, few studies have focused on the application of yeast and *Mitragyna speciosa* (Korth) Havil. in the goat production. Thus, we proposed that utilizing the complicated interactions between various feed additives, such as yeast and kratom leaf, may result in positive additive or synergistic effects that could increase animal production.

Therefore, the objectives of this study were to evaluate the effects of *Saccharomyces cerevisiae*, *Mitragyna speciosa* (Korth) Havil. and their combination on the digestibility of the feed, rumen fermentation, hematological, and nitrogen balance in Thai Native-Anglo Nubian crossbred goats.

## MATERIALS AND METHODS

### Animal care

The study was conducted at the Prince of Songkla University Laboratory of Animal Nutrition and Experimental Farm. The Institutional Animal Care and Use Committee of Prince of Songkla University gave its approval and authorization to all protocols and procedures (approval code: AG012/2022).

### Preparing dried kratom leaves and yeast

The green vein type fresh kratom leaves of *M. speciosa* Korth (Rubiaceae) were gathered in Tambon Namphu, Ban Na San District, Surat Thani Province, Thailand in October 2020, where the herbarium vouchers (PSU No. 012821) have been stored. The Department of Pharmacognosy and Pharmaceutical Botany, Faculty of Pharmaceutical Sciences, Prince of Songkla University, Songkhla, Thailand has verified the authenticity of the plant material. PSU No. 10/2563 was authorized by the Thai Ministry of Agriculture to solely use plant materials for research [9] and approved the method of keeping kratom leaves safe before feeding them to animals. Crabtree-negative yeasts, *Candida tropicalis* KKU20 (*C. tropicalis* KKU20; CBS 94^T^ (U45749) obtained from the Department of Animal Science, Faculty of Agriculture, Khon Kaen University (Khon Kaen, Thailand) and had a content of ≥1.15×10^13^ cfu/g) [10].

### Animals, design, treatments, and management

Four male Thai Native×Anglo Nubian crossbred goats were obtained from Experimental Goat Farm of the Prince of Songkla University, in Hat Yai City, Songkhla Province, Thailand, 90110. These goats aged 8±1 months with BW of 20±0.13 kg were randomly assigned to a 4×4 Latin square experiment with a 2×2 factorial arrangement of treatments and assigned to the effects of 2 levels of yeast (Y) (Y, 0 and 0.5g/kg dry matter [DM]) and 2 levels of DKTL (DKTL, 0 and 4.44 g/kg DM). The animals were as follows: T1 = control group containing 0Y+0DKTL, T2 = 0Y+4.44DKTL, T3 = 0.5Y+0DKTL, and T4 = 0.5Y+4.44DKTL. The diets were created to achieve a daily gain of 100 g in accordance with NRC [11] requirements.

All goats were kept individually in a ventilated metabolism crate (0.115×0.95 m) in well-ventilated sheds where water and mineral salt were always available. These goats were fed twice a day at 0700 am and 1700 pm and had free access to water. Vaccinations and other preventative precautions were carried out before the trial’s start in accordance with the Institutional Animal Care and Use Committee of the Prince of Songkla University. The experiment was carried out over four 21-day intervals, with the first 14 days dedicated to adjusting to and measuring feed consumption. Samples of feed, urine, and excrement from the previous 7 days were collected for chemical analysis. All animals were given their own total mixed rations (TMR) consisting of 30% Pangola grass hay (as roughage sources) and 70% concentrated diet. The ingredients and nutritional composition of TMR and Pangola grass hay (PGH) are shown in Table 1.

### Feed and fecal sampling procedures

Daily records of the amount of feed provided and the number of samples were kept throughout the experiment. Using the entire collection method, samples of feces, feed, and refusals were taken from each individual goat at the conclusion of each period. Using AOAC [12] method, DM, ash, ether extract (EE), and crude protein (CP) were assessed. According to Van Soest et al [13] acid detergent fiber (ADF) and neutral detergent fiber (NDF) were identified. By employing the techniques outlined by Jamil et al [14], alkaloids were identified and extracted from the plant. The modified vanillin-HCL technique was used to assess the DKTL samples for CT and SP [15]. A measurement was made with an atomic absorption spectrophotometer. The chemical composition of the DKTL is shown in Table 2.

### Urine sampling method

To keep the final pH below three and prevent nitrogen (N) extinction, whole urine was collected on the same days as feces in a plastic container treated with 10% H_2_SO_4_. Using the AOAC [16] total N measurement method, urine samples were collected at roughly 100 mL of total volume, frozen, and pooled at the conclusion of each session. The number of microbial purines absorbed (*x* mmol/d) corresponding to the purine derivatives excreted (*Y* mmol/d) was calculated according to Chen et al [17] as follows:


$$\text{Y}=0.84x+(0.15{\text{BW}}^{0.75}e^{-0.25}x)$$

Where Y is the excretion of purine derivatives (mmol/d); *x* is the microbial purines absorbed (mmol/d); BW is the body weight. Microbial N supplied to the small intestine was calculated from microbial purine absorbed (*x*) according to the equation of Chen and Gomes [18]:


$$\text{Microbial N }(\text{g}/\text{d})=\frac{70x}{0.83\times 0.116\times 1,000}$$

### Rumen fluid sampling procedures

On the final day of the data collection period, rumen fluid samples were taken at 0- and 4-hours following feeding. Each time, rumen fluid was collected from the center of the rumen using a stomach tube connected to a vacuum pump. The pH and temperature of the rumen fluid were measured right away using a portable temperature and pH meter (HANNA Instruments HI 98153 microcomputer pH meter, Kallang Avenue, Singapore). The rumen fluid samples were then filtered through four layers of cheesecloth. To stop the microbial activity fermentation process, 45 mL of rumen fluid were collected and kept in a plastic bottle with 5 mL of sulfuric acid solution (1 M). The mixture was centrifuged for 15 minutes at 16,000×g (Table Top Centrifuge PLC-02, Enfield, CT, USA). The supernatant was analyzed for ammonia nitrogen (NH_3_-N) using a Kjeltech Auto 1030 Analyzer (Foss, Hilleroed, Denmark), and volatile fatty acids (VFAs) were assessed using high-pressure liquid chromatography (HPLC; ETL Testing Laboratories, Inc., Cortland, NY, USA) [19]. According to the Moss et al [20] equation, ruminal CH_4_ can be estimated using VFA proportions as follows: CH_4_ production = 0.45 (acetate)× 0.275 (propionate)+0.4 (butyrate).

Another sample about 20 mL of ruminal fluid was collected to analyze for bacteria, protozoa, and zoospores and one mL was added to a formalin solution. In this procedure 1 mL of ruminal fluid from 20 mL was kept in 9 mL of 10% formaldehyde. Samples were kept chilled after dilution. For protozoa counts, a Sedgewick-Rafter chamber (S.I Scientific Supplies Co., Ltd., Bangkok, Thailand) was used with a cover slip. Finally, to find the average per square, 25 large squares were counted randomly and divided the number of protozoa counted by 25. For bacterial and fungi counts, a Petroff-Hausser chamber (Xinxiang Vic Science & Education Co., Ltd., Henan, China) was used [21] and to enumerate the bacteria, protozoa, and fungi according to Galyean’s [22] procedures using a microscope (Olympus BX51TRF, No. 2B04492, Olympus optical Co. Ltd., Tokyo, Japan).

### Blood sampling analysis

Blood samples (about 10 mL) were taken from the jugular vein at 0 and 4 h post feeding in tubes containing 12 mg of ethylenediaminetetraacetic acid on the last day of the data collection period. The plasma was kept at −20°C until it was analyzed blood urea nitrogen (BUN).

### Statistical analyses

All data were conducted using the general linear model (GLM) procedure of SAS (SAS Inst. Inc., Carry, NC, USA). The model used was: Model, Y*_ijk_* = μ+A*_i_*+P*_j_*+Y*_k_*+K*_l_*+YK*_kl_*+E*_ijkl_*, where Y*_ijkl_* = nutrient intake or rumen fermentation values; μ = overall mean; A*_i_* = effect of animal; P*_j_* = effect of period; Y*_k_* = effect of level of Y; K*_l_* = effect of level of DKTL; YK*_kl_* = effect of interaction of level of Y and DKTL; E*_ijkl_* = error of the term. Treatment means were statistically compared using Duncan’s multiple range test [23] to identify differences between means. Significant differences were declared if p<0.05.

All data were statistically analyzed in a 2×2 factorial arrangement in a 4×4 Latin square design by analysis of variance using GLM procedure of SAS (SAS Inst. Inc., USA). The results are presented as mean values and standard error of the means. The statistical model included supplementation of Y, supplementation of DKTL and interactions between Y and DKTL supplementation.

## RESULTS AND DISCUSSION

### Chemical composition

The feed ingredients and chemical compositions composed of basal diet (TMR), PGH, and DKTL are summarized in Table 1 and 2. The TMR, PGH, and DKTL contained 162, 59, and 21 g/kg CP on DM basis, respectively. Additionally, DKTL consisted of 91.5% DM, 87.0% OM, 13.5% ash, 19.8% NDF and 16.8% ADF. Moreover, secondary plant metabolites, such as alkaloids, particularly mitragynine, total phenolics, total CT, total SP content, and total flavonoids content in DKTL were 41, 41, 83, 52, and 112 g/kg DM, respectively. Macro minerals were Ca, 8 g; P, 2 g; K, 15 g; Mg, 3 g; S, 13 g; and 0.1 g Na, respectively. These variations may be a result of different materials, growing locations, and plant factory processing. However, the chemical content of Kratom leaves may vary depending on factors such as the environment in which the plant growth, season, light intensity, and weather conditions, etc. [24,25].

### Feed intake and nutrient digestibility in goats

There were no Y×DKTL interactions (p>0.05) with respect to feed intake, nutrient intake, apparent digestibility, digestible nutrient intake, and estimated energy intake (Tables 3, 4). However, the goats fed T4 (0.5Y+4.44DKTL) had the greatest total DMI. *Candida tropicalis* can improve fibrous material digestion, antioxidant function, and rumen microbial activity [26]. Furthermore, *Candida tropicalis* on biomass hydrolysate with ammonium sulfate as a nitrogen source [27] increased yield performance by boosting DM uptake [28]. Habeeb [29] found that yeast cell wall products increased the palatability of feed. 5′-nucleotide and glutamate can affect the amount eaten and the cell wall concentration was effective in stimulating microbial activity. Yeast fermentation with raw materials and then feeding to ruminants often results in increasing the fermentation efficiency in the rumen, digestion and number of microorganisms that allow animals to obtain more protein microorganisms [30]. Nutrients intake of organic matter (OM), CP, EE, NDF and ADF were not significantly different (p>0.05). Similarly, Chanjula et al [7] studied DKTL in goat and found no difference in dietary intake in terms of OMI, CPI, NDFI and ADFI. Dias et al [31] investigated the effect of yeast supplementation in low starch and high starch levels in the diet on rumen fermentation and digestibility in dairy cows. It was found that yeast supplementation affected total protein digestibility in cows fed the diet. In addition, García et al [32] studied the effects of yeast and monensin supplementation in sheep and found no effect on DM digestibility.

The phytochemical or anti-nutritional chemicals such as mitragynine, CT, SP, and phenolic acids contained in the kratom leaves affected the total feed intake and digestibility. Kratom leaf supplementation increased by more than 4.44 g/h/d similar to the study of Sultana et al [33]; Su and Chen [34] reported that the CT and SP content was content in dried moringa leaves reduces nutrient digestibility. *Sanguisorba officinalis* supplementation 100 mg was found to decrease nutrient digestibility [35]. Mitragynine binds to many receptors such as opioid, serotonin and adrenergic receptors, etc., resulting in pharmacological properties such as reducing acid secretion, intestinal peristalsis, appetite and anti-inflammatory, etc. Especially high levels of DKTL supplementation may reduce intestinal peristalsis. However, the exact mechanism is still unknown which requires further detailed study.

### Ruminal fermentation and blood urea nitrogen

The rumen fermentation is presented in Table 5. The temperature of the rumen did not differ between the groups (p>0.05). The ruminal pH parameters changed when supplemented with yeast; the value remained stable between 6.70 and 6.81. Yeast cells affected the ruminal pH compared to control. In all cases the pH values remained above 6.5, the physiological range of suitable for the microorganisms fermentation (cellulolytic bacteria) and protein digestion [36].

Ruminal NH_3_-N concentration was not affected by yeast and DKLT supplements, which is consistent with other studies [7]. Our findings demonstrated that DKTL feeding produced improved rumen fermentation. Targeted additions of CT and SP-containing feedstuffs may improve rumen efficiency by maintaining a higher pH and promoting microbial protein production [37]. Supplementation with DKTL led to reduced levels of NH_3_-N and BUN when compared to the control group. The CT and SP in DKTL, which may form protein complexes can efficiently increase bypass protein.

DKTL was added to the diet, and this resulted in a decrease in BUN concentrations at 0 hours after feeding. To determine whether there was a connection between rumen NH_3_-N and CP intake, blood BUN was also measured. All the crucial indications of rumen stability and function are the BUN levels [38]. BUN levels were a 16.28 to 20.57 mg/dL range. However, Anantasook et al [39] and Patra and Yu [40] found that CT produced a protein-tannin complex that limited the availability of ruminal breakdown dietary protein and decreased NH_3_-N production, however, due to the high level of supplementation the results were inconsistent with the present study. Furthermore, tannin binding made more protein unavailable for bacterial conversion to NH_3_-N. There is a lot of proof that DKTL affects the rumen’s microbial fermentation [41–43]. Moreover, reducing NH_3_-N levels in the rumen resulted in decreased BUN values.

### Volatile fatty acid and methane production

The VFA profile and methane production are shown in Table 6. The total VFA showed no interaction between the DKTL level or yeast (p>0.05). Total VFA increased at 4h after feeding when yeast was supplemented (p<0.05). The concentration of C2 decreased upon an interaction in the DKTL and yeast, due to the increased ratio of C3 synthesis. Furthermore, concentration of C3 increased when goat was fed DKTL and yeast (p<0.05). The mechanisms producing a higher VFAs concentration in yeast-fed animals appeared to be associated with an increased activity of the anaerobic microflora. By increasing rumen propionic, reducing protozoa, and consequently lowering methane production, feed containing CT and SP significantly improved rumen fermentation [44,45]. The anticipated shift from CH_4_ to H_2_, which is advantageous for the host’s energy supply, caused the VFA profile to go from C2 to C3 [46].

Methane production at 0 and 4 h after feeding with the Y level of 0.5 and DKTL 4.44 g/kg DM decreased when compared to other groups. The average was between 20.33–29.42 and 21.63–29.06 g/d. Rumen CH_4_ production was strongly related to microbial fermentation. Tannin can reduce CH_4_ emission by affecting rumen H+ exchange capacity and can also affect all the end fermentation characteristics. Reduction in CH_4_ can be achieved indirectly by decreasing protozoal abundance [47]. Moreover, CH_4_ production was consequently decreased by 0.5Y+4.44DKTL supplementation. This could be the result of the suppression of rumen protozoal number and methanogens. Similarly, in a study by Phesatcha et al [46], it was discovered that supplementing with *Mitragyna speciosa* Korth leaf pellets can influence ruminal fermentation by reducing C2 concentration and mitigating CH_4_ production, consequently leading to an increase in C3 concentration.

### N metabolism and utilization

**Figure 1:**
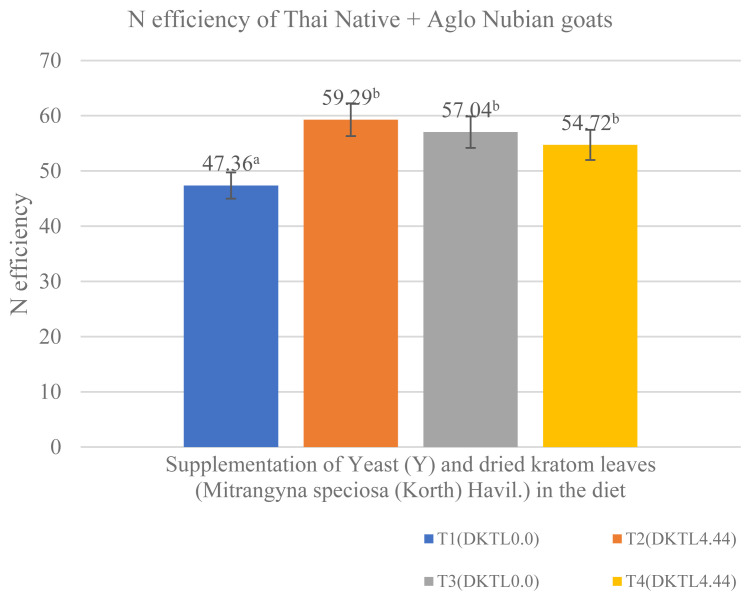
Effects of feeding combinations of Yeast (Y) and dried kratom leaves (*Mitrangyna speciosa* (Korth) Havi.) (DKTL) supplementation on N efficiency. ^a,b^ Means with different superscripts are significantly different.

There were no interaction effects on N balance and microbial protein between Y and DKTL fed to the animals (p>0.05), except N efficiency and urine litres per day (p<0.05) (Table 7). The N efficiency was the highest (59.29%) when feeding DKTL at 4.44% without Y (Figure 1). Supplementation Y and with various DKTL levels affected the percentage of N intake fecal and N intake absorbed. Increased levels of Y supplementation at 0.5% with high levels of DKTL at 4.44% in goat diet decreased the fecal N intake to 23.07% and increased the absorbed N intake to 76.93%. Y and DKTL showed an interaction on N. The most important metric for assessing ruminants’ protein nutrient status is efficiency. Similarly, Viennasay et al [48] observed that increasing the digestibility of CP led to a rise in retained nitrogen. Increased CP and CT consumption increased protein transport from the rumen to the small intestine [49]. Current study found that the nitrogen efficiency was positive in accordance with the total feed intake, probably due to the amount of digestibility and the nutrients intake.

### Ruminal microorganism population

Feeding Y with DKTL changed purine derivative (allatoin, purine derivative [PD] excretion and PD absorption) and microbial protein synthesis which was higher with Y supplementation at 0.5% of Y (p<0.05). The bacterial population was between (1.53 – 2.11×10^9^ cell/mL) and the fungus (0.96 – 1.37×10^6^ cell/mL), respectively (Table 8).

Goats fed Y with DKTL also showed no effect on bacterial and fungal populations at various feeding times and mean values. However, the protozoal population decreased when feeding DKTL to animals after 0 and 4 h morning post feeding. The reduction in protozoa populations caused by DKTL supplementation (p<0.01) may be explained by the CT and SP in the DKTL. CT and SP binds portions of the cell membrane of protozoa and methanogens [50]. It has a considerable impact on methanogenic archaea in the rumen [46]. It has the capacity to prevent the transfer of hydrogen while also limiting the development of methanogens.

With high concentrate-based diets, one of the most frequently observed effects of live yeast supplementation is an increase in the number and activity of the bacterial population in the rumen, which appears to improve the rumen’s capacity to metabolize lactic acid and control ruminal pH [51]. To increase production levels in domesticated ruminants, diets rich in cereal grains are frequently offered.

## CONCLUSION

Effects of feeding combinations of Y and DKTL supplementation on feed showed no interaction effect (Y×DKTL) on feed intake, rumen fermentation, bacterial and fungi population. Protozoal populations were lower in the group that was supplemented with DKTL at 4.44 g/kg. The DM related to synthetic CH4 was reduced. In addition, Y and DKTL supplementation should be studied in fattening and milking goats at different stages. Including analyzing the economic returns incurred under farm conditions and its dietary effect to total intake, nutrient intake, nutrient digestibility, and N efficiency.

## Figures and Tables

**Table 1:** Ingredients and nutrition of total mixed ration (TMR) as the basic diet and pangola grass hay (PGH) fed to the goats

Item (g/kg)	TMR^1)^		DKTL

	Concentrate diet	Roughage source	
Pangola grass hay	-	300	
Ground corn	362	-	
Soybean meal	227	-	
Fish meal	5	-	
Leucaena leave meal	40	-	
Molasses	50	-	
Dicalcium phosphate	3	-	
Salt	3	-	
Mineral and vitamin mix^2)^	10	-	
Chemical composition			
Dry matter	880	910	255
Ash	50	37	41
Organic matter	950	964	959
Crude protein	162	59	21
Neutral detergent fiber	482	820	445
Acid detergent fiber	243	452	273
Acid detergent lignin	44	380	85
Ether extract	23	13	17
Gross energy (kcal/kg DM)	4,475	2,194	4,630

1) TMR diet was divided into four treatments depending on Y and DKTL supplementation level: T1 = supplemented 0Y-0DKTL; T20 = 0Y-4.44DKTL; T3 = 0.5Y-0DKTL; T4 = 0.5Y-4.44DKTL (g/kg DM in TMR).

2) Minerals and vitamins (each kg contains): Vitamin A: 10,000,000 IU; Vitamin E: 70,000 IU; Vitamin D: 1,600,000 IU; Fe: 50 g; Zn: 40 g; Mn: 40 g; Co: 0.1 g; Cu: 10 g; Se: 0.1 g; I: 0.5 g.

All data was measured except for ME.

Note: Components of MIP: Bacillus subtilis ≥ 5.0×10^7^ CFU/g, Bacillus licheniformis ≥ 1.0×10^8^ CFU/g, yeast ≥1.0×10^8^ CFU/g; components of CYP: Total Saccharomyces cerevisiae cells (DM) ≥10×10^8^ CFU/g, cellulose activity ≥3,000 U/g, xylanase activity ≥2,000 U/g, β-glucanase activity ≥15,000 U/g, amylase activity ≥20,000 U/g, protease activity ≥2,000 U/g and some other fermented metabolites.

**Table 2:** Chemical composition and nutritive values of dried kratom leaves (DKTL) used in the experimental diets (on a dry matter basis) for goats

Parameters	DKTL^1)^
Alkaloid profile (%)	
Mitragynine	4.14
Paynantheine	0.59
Speciogynine	0.26
Total condensed tannin content	8.28
Total saponin content	5.21
Flavonoids	11.24
Phenolic acids	4.1
Antioxidant activity	
DPPH (IC50 (mg/mL)	1.04
FRAP (%)	3.98
Mineral profile^2)^	
Ca (%)	0.84
P (%)	0.2
K (%)	1.53
Mg (%)	0.3
S (%)	1.26
Na (%)	0.01
Fe (ppm)	80.67
Cu (ppm)	11.54
Mn (ppm)	1862.3
Zn (ppm)	32.14
B (ppm)	69.71
Cr (ppm)	3.23

DKTL, dried kratom leaves; DPPH, 2,2-diphenyl-1-picrylhydrazyl; FRAP, ferric reducing antioxidant power.

1) Tambon Namphu, Ban Na San, Surat Thani Province, Thailand.

2) Ca, calcium; P, phosphorus; K, potassium; Mg, magnesium; S, sulfur; Na, sodium; Fe, iron; Cu, copper; Mn, manganese; Zn, zinc; Cr, chromium.

**Table 3:** Effects of feeding combinations of yeast and dried kratom leaves (*Mitrangyna speciosa* (Korth) Havi.) supplementation on feed intake and nutrient intake in goats

Items	Treatments^1)^				SEM	p-value^2)^		

	0.0Y		0.5Y					
		
	0DKTL	4.44DKTL	0DKTL	4.44DKTL		Y	DKTL	Y×DKTL
DMI (kg/d)								
Total DMI (kg/d)	0.751^b^	0.771^ab^	0.774^ab^	0.830^a^	0.01	0.04	0.10	0.12
DMI (% BW)	3.34^b^	3.56^ab^	3.65^ab^	3.72^a^	0.08	0.04	0.16	0.09
DMI (g/kg W^0.75^)	72.63^b^	76.56^ab^	78.26^ab^	80.73^a^	1.93	0.04	0.14	0.11
Nutrient intake (kg/d)								
OMI	0.709	0.728	0.731	0.784	0.01	0.08	0.11	0.40
CPI	0.122	0.125	0.125	0.134	0.003	0.08	0.10	0.40
EEI	0.017	0.017	0.018	0.019	0.0004	0.12	0.12	0.47
NDFI	0.362	0.371	0.373	0.400	0.009	0.09	0.10	0.39
ADFI	0.182	0.187	0.188	0.201	0.004	0.08	0.10	0.39

SEM, standard error of the mean; DMI, dry matter intake; BW, body weight; BW^0.75^, metabolic body weight; OMI, organic matter intake; CPI, crude protein intake; EEI, ether extract intake; NDFI, neutral detergent fiber intake; ADFI, acid detergent fiber intake; TMR, total mixed rations.

1) T1, supplemented 0Y-0DKTL; T2, 0Y-4.44DKTL; T3, 0.5Y-0DKTL; T4, 0.5Y-4.44DKTL (g/kg DM in TMR).

2) Y, yeast; DKTL, dried kratom leaves.

a,b Means with different superscripts within the same row are significantly different (p<0.05).

**Table 4:** Effects of feeding combinations of yeast (Y) and dried kratom leaves (*Mitrangyna speciosa* (Korth) Havi.) (DKTL) supplementation on apparent digestibility and digestible nutrient intake of nutrients in goats

Item	Treatments^1)^				SEM	p-value^2)^		

	0.0Y		0.5Y					
		
	0DKTL	4.44DKTL	0DKTL	4.44DKTL		Y	DKTL	Y×DKTL
Apparent digestibility (%)								
DM	75.48^b^	77.32^a^	77.35^a^	78.53^a^	0.36	0.005	0.006	0.39
Ash	45.86^b^	50.72^ab^	53.02^ab^	57.53^a^	2.47	0.03	0.10	0.94
OM	77.19^b^	78.85^a^	78.73^a^	79.74^a^	0.36	0.01	0.01	0.40
CP	74.09^b^	76.10^ab^	75.74^a^	76.95^a^	0.50	0.04	0.01	0.46
NDF	67.41^b^	70.41^a^	70.35^a^	71.98^a^	0.63	0.01	0.01	0.32
ADF	59.70^b^	61.68^b^	62.23^ab^	65.14^a^	0.88	0.01	0.03	0.62
Digestible nutrient intake (g/d)								
DOM^3)^	0.547^b^	0.574^ab^	0.576^ab^	0.626^a^	0.01	0.04	0.05	0.48
DCP	0.090^b^	0.095^ab^	0.095^ab^	0.103^a^	0.002	0.006	0.006	0.57
DNDF	0.244^b^	0.261^b^	0.262^b^	0.288^a^	0.007	0.01	0.02	0.59
DADF	0.109^b^	0.115^b^	0.117^b^	0.131^a^	0.003	0.01	0.02	0.31
Estimated energy intake								
ME (Mcal/d)	2.08^b^	2.18^ab^	2.19^ab^	2.38^a^	0.05	0.03	0.04	0.45
ME (Mcal/kg/DM)	2.77^b^	2.83^a^	2.83^a^	2.86^a^	0.01	0.01	0.01	0.36

SEM, standard error of the mean; DM, dry matter; OM, organic matter; CP, crude protein; NDF, neutral detergent fiber; ADF, acid detergent fiber; DOM, digestible organic matter; DCP, digestible crude protein; DNDF, digestible neutral detergent fiber; DADF, digestible acid detergent fiber; ME, metabolizable energy.

1) T1 = supplemented 0Y-0DKTL; T2 = 0Y-4.44DKTL; T3 = 0.5Y-0DKTL; T4 = 0.5Y-4.44DKTL (g/kg DM in TMR).

2) Y, yeast; DKTL, dried kratom leaves.

3) 1 kg DOM = 3.8 Mcal ME/kg (Kearl, 1982).

a,b Means with different superscripts within the same row are significantly different (p<0.05).

**Table 5:** Effects of feeding combinations of yeast (Y) and dried kratom leaves (*Mitrangyna speciosa* (Korth) Havil.) (DKTL) supplementation on ruminal fermentation in goats

Item	Treatments^1)^				SEM	p-value		

	0.0Y		0.5Y					
		
	0DKTL	4.44DKTL	0DKTL	4.44DKTL		Y	DKTL	Y×DKTL
Temperature (°C)								
0 h-post feeding	38.57	38.85	39.07	39.15	0.27	0.20	0.55	0.73
4 h	38.35	39.00	38.95	38.75	0.21	0.44	0.33	0.09
Mean	38.46	38.92	39.01	38.95	0.13	0.08	0.19	0.10
Ruminal pH								
0 h-post feeding	6.81^b^	6.85^b^	7.00^a^	6.93^a^	0.05	0.05	0.85	0.38
4 h	6.58	6.59	6.63	6.66	0.03	0.13	0.57	0.71
Mean	6.70^b^	6.72^b^	6.81^a^	6.80^a^	0.03	0.04	0.89	0.61
Ruminal NH_3_-N (mg/dL)								
0 h-post feeding	17.93	21.15	20.79	21.15	3.10	0.66	0.58	0.66
4 h	21.15	21.86	23.29	22.82	2.57	0.56	0.96	0.82
Mean	19.54	21.50	22.04	21.99	2.53	0.57	0.71	0.70
BUN (mg/dL)								
0 h-post feeding	20.84^a^	16.17^b^	17.08^b^	16.60^b^	0.94	0.12	0.03	0.06
4 h	20.29	16.40	18.23	17.30	1.82	0.76	0.23	0.44
Mean	20.57	16.28	17.65	16.95	1.24	0.40	0.09	0.20

SEM, standard error of the mean; NH_3_-N, ammonia nitrogen; BUN, blood urea nitrogen.

1) T1 = supplemented 0Y-0DKTL; T2 = 0Y-4.44DKTL; T3 = 0.5Y-0DKTL; T4 = 0.5Y-4.44DKTL (g/kg DM in TMR).

a,b Means with different superscripts within the same row are significantly different (p<0.05).

**Table 6:** Effects of feeding combinations of yeast (Y) and dried kratom leaves (*Mitrangyna speciosa* (Korth) Havil.) (DKTL) supplementation on volatile fatty acid profiles in goats

Item	Treatments^1)^				SEM	p-value		

	0.0Y		0.5Y					
		
	0DKTL	4.44DKTL	0DKTL	4.44DKTL		Y	DKTL	Y×DKTL
Total VFA (mmol/L)								
0 h-post feeding	75.51	77.89	72.94	71.85	6.85	0.55	0.92	0.80
4 h	58.97^b^	62.51^b^	79.10^a^	74.36^a^	4.45	0.01	0.89	0.38
Mean	67.24	70.20	76.02	73.10	5.04	0.29	0.99	0.58
C2 %								
0 h-post feeding	63.93	58.98	57.95	57.12	2.69	0.19	0.32	0.47
4 h	64.77^a^	58.61^ab^	54.55^b^	54.54^b^	2.12	0.01	0.19	0.19
Mean	64.35^a^	58.80^b^	56.25^b^	55.83^b^	0.98	0.001	0.02	0.04
C3 %								
0 h-post feeding	20.15	23.77	25.45	27.94	2.63	0.12	0.28	0.83
4 h	19.12^b^	24.80^ab^	28.78^a^	31.59^a^	2.57	0.01	0.15	0.59
Mean	19.64^c^	24.29^cb^	27.11^cb^	29.77^a^	1.46	0.004	0.04	0.52
C4 %								
0 h-post feeding	13.68	14.69	13.90	12.27	2.10	0.62	0.88	0.55
4 h	13.82	13.92	14.64	11.20	1.51	0.55	0.31	0.28
Mean	13.75	14.31	14.27	11.73	0.70	0.19	0.20	0.06
%Other VFA^2)^								
0 h-post feeding	2.25	2.55	2.70	2.66	0.33	0.43	0.70	0.62
4 h	2.28	2.66	2.03	2.68	0.24	0.64	0.08	0.60
Mean	2.26	2.61	2.36	2.67	0.27	0.78	0.28	0.95
C2:C3								
0 h-post feeding	3.24	2.69	2.40	2.15	0.37	0.11	0.33	0.70
4 h	3.44^a^	2.46^ab^	2.02^b^	1.94^b^	0.30	0.01	0.12	0.18
Mean	3.34^a^	2.57^b^	2.21^b^	2.05^b^	0.18	0.004	0.04	0.15
C2+4:3								
0 h-post feeding	3.93	3.38	2.93	2.62	0.46	0.10	0.39	0.80
4 h	4.18^a^	3.04^ab^	2.55^b^	2.33^b^	0.37	0.02	0.12	0.27
Mean	4.06^a^	3.21^b^	2.74^b^	2.48^b^	0.22	0.004	0.05	0.24
CH_4_ (g/d)								
0 h-post feeding	28.70	25.88	24.64	22.93	1.90	0.11	0.27	0.78
4 h	29.42^a^	25.12^ab^	22.49^b^	20.33^b^	1.75	0.01	0.11	0.56
Mean	29.06^a^	25.50^b^	23.57^bc^	21.63^c^	1.02	0.003	0.03	0.45

SEM, standard error of the mean; VFA, volatile fatty acid; C2, acetic acid; C3, propionic acid; C4, butyric acid; CH_4_, methane.

1) T1 = supplemented 0Y-0DKTL; T2 = 0Y-4.44DKTL; T3 = 0.5Y-0DKTL; T4 = 0.5Y-4.44DKTL (g/kg DM in TMR).

2) Sum of isobutyrate, isovalerate, valerate, and caproate.

a–c Means with different superscripts within the same row are significantly different (p<0.05).

**Table 7:** Effects of feeding combinations of yeast (Y) and dried kratom leaves (*Mitrangyna speciosa* (Korth) Havi.) (DKTL) supplementation on N balance and microbial protein in goats

Item	Treatments^1)^				SEM	p-value		

	0.0Y		0.5Y					
		
	0DKTL	4.44DKTL	0DKTL	4.44DKTL		Y	DKTL	Y×DKTL
N balance (g/d)								
Intake	19.44	19.96	20.04	21.48	0.51	0.08	0.10	0.40
Fecal	5.03	4.76	4.85	4.95	0.10	0.99	0.44	0.12
Urine	5.23	3.33	3.75	4.88	0.69	0.96	0.59	0.07
Total N loss	10.27	8.10	8.60	9.82	0.66	0.96	0.50	0.40
Absorbed	14.40	15.20	15.19	16.54	0.46	0.06	0.06	0.57
Retained	9.17	11.86	11.44	11.66	0.75	0.10	0.21	0.15
% of N intake								
Fecal	25.93^a^	23.93^b^	24.28^ab^	23.07^b^	0.50	0.04	0.01	0.46
Urine	26.72	16.77	18.68	22.21	3.07	068	0.33	0.07
Absorbed	74.07^b^	76.07^a^	75.72^ab^	76.93^a^	0.50	0.04	0.01	0.46
N Efficiency	47.36^b^	59.29^a^	57.04^ab^	54.72^ab^	2.94	0.41	0.15	0.05
Urine (Litters/d)	0.530^a^	0.396^ab^	0.445^b^	0.550^ab^	0.04	0.47	0.75	0.03
Purine derivative (mmol/d)								
Allantoin (mmol/d)	12.97^ab^	11.09^b^	17.47^ab^	23.57^a^	3.06	0.03	0.51	0.24
PD excretion (mmol/d)	15.44^ab^	13.21^b^	20.79^ab^	28.06^a^	3.65	0.03	0.51	0.24
PD absorption (mmol/d)	18.30^ab^	15.64^b^	24.67^ab^	33.32^a^	4.35	0.03	0.51	0.24
Microbial N (g/d)	13.31^ab^	11.37^b^	17.94^ab^	24.22^a^	3.16	0.03	0.51	0.24
EMNS (g/kg)	36.73^ab^	30.51^b^	46.92^ab^	58.69^a^	7.14	0.03	0.71	0.25

SEM, standard error of the mean.

1) T1 = Supplemented 0Y-0DKTL; T2 = 0Y-4.44DKTL; T3 = 0.5Y-0DKTL; T4 = 0.5Y-4.44DKTL (g/kg DM in TMR).

EMNS, efficiency of microbial N synthesis = [MN(g/d)×1,000 (g)]/DOMR (g); where DOMR = DOMI×0.65, DOMR = digestible organic matter apparently fermented in the rumen and DOMI = digestible organic matter intake.

a,b Means with different superscripts within the same row are significantly different.

**Table 8:** Effects of feeding combinations of yeast (Y) and dried kratom leaves (*Mitrangyna speciosa* (Korth) Havi.) (DKTL) supplementation on the microorganism count in the rumen fluid of goats

Item	Treatments^1)^				SEM	p-value		

	0.0Y		0.5Y					
		
	0DKTL	4.44DKTL	0DKTL	4.44DKTL		Y	DKTL	Y×DKTL
Total direct counts (cell/mL)								
Bacteria (×10^9^)								
0 h-post feeding	1.40	1.60	1.66	2.05	0.16	0.07	0.11	0.59
4 h	1.61	2.01	1.97	2.18	0.25	0.33	0.27	0.71
Mean	1.53	1.81	1.82	2.11	0.16	0.13	0.14	0.94
Protozoa (×10^5^)								
0 h-post feeding	10.15^a^	7.85^b^	9.50^a^	7.05^b^	0.46	0.13	<0.01	1.02
4 h	10.45^a^	8.05^b^	10.72^a^	7.72^b^	0.50	1.02	<0.01	0.63
Mean	10.30^a^	7.95^b^	10.11^a^	7.38^b^	0.31	0.25	<0.01	0.71
Fungal zoospores (×10^6^)								
0 h-post feeding	1.04	1.29	1.26	1.35	0.19	0.48	0.39	0.69
4 h	0.88	1.26	1.10	1.39	0.20	0.42	0.14	0.80
Mean	0.96	1.28	1.18	1.37	0.18	0.43	0.22	0.74

SEM, standard error of the mean.

1) T1, supplemented 0Y-0DKTL; T2, 0Y-4.44DKTL; T3, 0.5Y-0DKTL; T4, 0.5Y-4.44DKTL (g/kg DM in TMR).

a,b Means with different superscripts within the same row are significantly different.
